# Heart vs. Brain in a Warzone: The Effects of War on Acute Cardiovascular and Neurological Emergencies

**DOI:** 10.3390/diagnostics15162081

**Published:** 2025-08-19

**Authors:** Vladimir Zeldetz, Sagi Shashar, Carlos Cafri, David Shamia, Tzachi Slutsky, Tal Peretz, Noa Fried Regev, Naif Abu Abed, Dan Schwarzfuchs

**Affiliations:** 1 Department of Emergency Medicine, Soroka University Medical Center, Beer Sheva 84101, Israel; vladimirz@clalit.org.il (V.Z.); tzachisl@clalit.org.il (T.S.); zamstein.tal@gmail.com (T.P.); friednoaa@gmail.com (N.F.R.); naifab@clalit.org.il (N.A.A.); dansch@clalit.org.il (D.S.); 2 Clinical Research Center, Faculty of Health Sciences, Soroka University Medical Center, Ben-Gurion University of the Negev, Beer Sheva 84101, Israel; 3 Department of Cardiology, Soroka University Medical Centre, Beer Sheva 84101, Israel; cafricar@clalit.org.il (C.C.); shamiada@clalit.org.il (D.S.); 4 Hospital Administration, Soroka University Medical Center, Beer Sheva 84101, Israel

**Keywords:** STEMI, ischemic stroke, wartime medicine, emergency care, health system resilience, treatment delay, crisis preparedness, patient behavior, symptom-to-door time, armed conflict

## Abstract

**Background**: Armed conflicts impose complex logistical and behavioral challenges on healthcare systems, particularly in managing acute conditions such as ST-elevation myocardial infarction (STEMI) and ischemic stroke. Although both diagnoses require timely intervention, their clinical pathways differ significantly. Few studies have systematically compared their management during active warfare, particularly within the warzone. **Methods**: This retrospective cohort study was conducted at Soroka University Medical Center (SUMC), the sole tertiary hospital in southern Israel and the main referral center for cardiovascular and neurological emergencies in the region. We included all adult patients (≥18 years) admitted with new-onset STEMI or ischemic stroke during three-month periods of wartime (October–December 2023) and matched routine periods in 2021 and 2022. Patients with in-hospital events, inter-hospital transfers, or foreign citizenship were excluded. Data on demographics, comorbidities, arrival characteristics, treatment timelines, and outcomes were extracted from electronic medical records. Categorical variables were compared using Chi-squared or Fisher’s exact test, and continuous variables using *t*-tests or Mann–Whitney U tests, as appropriate. Multivariable logistic and linear regression models were adjusted for age, sex, Charlson Comorbidity Index (CCI), and mode of arrival. Interaction terms assessed whether wartime modified the associations differently for STEMI and stroke. **Results**: A total of 410 patients were included (193 with STEMI and 217 with stroke). Patients with STEMI were significantly more likely to arrive by self-transport during the war (38, 57.6% vs. 32, 25.2%, *p* < 0.001) and had higher rates of late arrival beyond 12 h (19, 28.8% vs. 13, 10.2%, *p* = 0.002). These findings support the conclusion that patients were more prone to delayed and unstructured presentations during a crisis. In contrast, patients with stroke showed a reduction of 354 min in symptom-to-door times during the war [median 246 (30–4320 range) vs. 600 min (12–2329 range), *p* = 0.026]. Regression models revealed longer delays for stroke vs. STEMI in routine settings [β = 543.07 min (239.68–846.47 95% CI), *p* < 0.001], along with significantly lower in-hospital (OR = 0.39, 95% CI= 0.15–0.97, *p* = 0.05) and 30-day mortality (OR = 0.43, 95% CI= 0.19–0.94, *p* = 0.04). However, these differences were no longer significant during wartime. Patients with STEMI showed a trend toward lower 180-day mortality during the war (OR = 0.33, 95% CI = 0.09–0.99; *p* = 0.07), although this difference did not reach statistical significance. **Conclusions**: During wartime, patients with stroke arrived earlier and in greater numbers, while patients with STEMI showed reduced admissions and delayed, self-initiated transport. Despite these shifts, treatment timelines and short-term outcomes were maintained. These diagnosis-specific patterns highlight the importance of reinforcing EMS access for STEMI and preserving centralized protocol-based coordination for stroke during crises.

## 1. Introduction

Crises such as wars and pandemics impose substantial burdens on healthcare systems, particularly during time-sensitive emergencies. These disruptions affect not only hospital capacity but also health-seeking behaviors, ambulance logistics, and the speed of clinical decision-making. Among the most vulnerable conditions in such settings are ST-elevation myocardial infarction (STEMI) and ischemic stroke, both of which require rapid diagnosis and treatment to minimize irreversible damage and reduce mortality [1,2]. Studies have described how large-scale crises—such as wars, natural disasters, and pandemics—disrupt the care of acute conditions, particularly those requiring timely intervention such as STEMI and stroke [3,4]. During such events, admissions for STEMI often decline sharply, and delays in reperfusion have been widely reported [5]. These trends have been attributed to patient fear, reduced accessibility to emergency services, and system-level overload [6]. In contrast, the impact on stroke care has been more variable: some centers have documented reductions in imaging and thrombolysis, while others have maintained stable acute stroke pathways, especially in systems with centralized protocols and organized networks [7,8,9,10].

Although both STEMI and stroke are medical emergencies, their diagnostic and logistical pathways differ. STEMI care often involves direct EMS triage to catheterization labs based on ECG findings [11], while stroke pathways typically require ED-based imaging and neurologic consultation [12]. Because their workflows rely on different system components—prehospital triage versus in-hospital diagnostics—comparing them enables the isolation of the stages in the emergency chain that are most sensitive to crisis-related disruptions. While most studies have focused on either STEMI or stroke independently during crises, few have directly compared their management, particularly during the COVID-19 pandemic. For example, studies have shown that while both conditions were affected, stroke care experienced more pronounced diagnostic delays, whereas STEMI workflows were relatively preserved [13,14]. These findings support the hypothesis that systemic disruptions affect acute care pathways differently depending on diagnosis. However, to our knowledge, no prior study has systematically compared STEMI and stroke presentations and outcomes during an active armed conflict, particularly from within the warzone itself [7,15]. This study addresses this gap and provides complementary insights to those gained during the pandemic, highlighting how different types of crises may influence emergency care pathways through distinct mechanisms.

This study addresses this gap by examining acute STEMI and stroke care at Soroka University Medical Center (SUMC) during the 2023 Israel–Hamas war, a conflict in which the southern region of Israel, and specifically the hospital’s catchment area, came under continuous rocket fire [4,16,17,18]. Unlike previous analyses from unaffected metropolitan centers, this setting provides a unique opportunity to study health system performance under real-time threat. By comparing patient characteristics, arrival patterns, treatment delays, and outcomes between routine and wartime periods for both conditions, this study seeks to identify diagnosis-specific strengths and weaknesses under duress. These findings may inform future national preparedness policies, particularly in regions facing ongoing or recurrent security threats.

## 2. Methods

### 2.1. Study Design and Population

This retrospective cohort study was conducted at SUMC, the only tertiary care center in southern Israel and a primary referral center for cardiovascular and neurological emergencies in the Negev region. The study included all adult patients (age ≥ 18 years) who were admitted with either STEMI or acute ischemic stroke during defined three-month periods of wartime and routine conditions. Specifically, we examined three periods: (1) wartime—7 October to 31 December 2023, during the Israel–Hamas conflict; (2) two corresponding routine periods during the same calendar months in 2022 and 2021.

Only patients with a confirmed diagnosis of new-onset STEMI or stroke who presented from the community were included. Diagnoses were made in the emergency department using clinical evaluation and supporting diagnostic tools: stroke was diagnosed by neurologists and confirmed by CT interpreted by radiologists, and STEMI was confirmed based on electrocardiographic findings and clinical presentation. ICD-9 codes recorded at discharge were used for identifying cases. Patients were excluded if the event occurred during hospitalization for other causes, if they were transferred from other hospitals, or if they had foreign citizenship, as complete follow-up data were not reliably available. Symptom onset time was determined based on patient or accompanying person reports at admission.

### 2.2. Data Collection

Data were extracted from the institutional electronic medical records and included demographics (age, sex, and ethnicity), comorbidities (diabetes mellitus, hypertension, dyslipidemia, and ischemic heart disease), Charlson Comorbidity Index (CCI), mode of arrival (self, ambulance, or referral from emergency center), timing metrics (symptom-to-door, door-to-CT or needle, and door-to-treatment), and clinical outcomes. Late arrival was defined as arrival beyond 12 h after symptom onset. Stroke patients were also categorized according to treatment type: no reperfusion therapy, systemic thrombolysis, cerebral catheterization, or both.

The data were de-identified and handled according to ethical standards. The study was approved by the Institutional Helsinki Review Board of SUMC (0298-23-SOR). The requirement for informed consent was waived due to the retrospective nature of the study. No missing data were present for the variables included in this analysis, as all records were manually extracted and cross-validated by multiple physicians.

### 2.3. Outcomes

Primary outcomes included time intervals from symptom onset to arrival and to treatment, in-hospital mortality, and mortality at 30, 90, 180, and 365 days post-admission. Secondary outcomes included late arrival and length of stay.

### 2.4. Statistical Analysis

Categorical variables are reported as frequencies and percentages, and continuous variables were assessed for normality using the Shapiro-Wilk test and visualized using histograms. Normally distributed variables are expressed as means with standard deviations (SD), while non-normally distributed variables are presented as medians with interquartile ranges or full ranges, as appropriate.

Comparisons were made across two axes: (1) between wartime and routine periods within each diagnosis group (STEMI and stroke), (2) between STEMI and stroke during each period. Categorical variables were compared using the chi-squared test or Fisher’s exact test, and quantitative variables were compared using the Student’s *t*-test or Mann-Whitney U test, as appropriate.

Multivariable regression models were used to examine the associations between diagnosis (STEMI vs. stroke), period (wartime vs. routine), and clinical outcomes. In addition to evaluating the main effects, we tested for interaction between diagnosis and period to assess whether the effect of wartime conditions on outcomes differed between the two conditions. Continuous outcomes (e.g., symptom-to-door time) were analyzed using linear regression, and binary outcomes (e.g., mortality at various time points) were analyzed using logistic regression. All models were adjusted for age, sex, Charlson Comorbidity Index (CCI), and mode of arrival. Interaction terms were included in these models.

All statistical analyses were performed using RStudio version 2024.04.2 and Python version 3.8.5. A two-sided *p*-value of <0.05 was considered to be statistically significant. Because the study retrospectively included all eligible cases from the defined periods, no a priori sample size calculation was conducted. Nonetheless, the sample size was sufficient to detect several statistically significant differences in key outcomes.

## 3. Results

### 3.1. Study Population

The study cohort comprised 410 patients diagnosed with STEMI or acute ischemic stroke during periods of routine care and wartime. Of these, 217 patients (52.9%) presented with stroke and 193 (47.1%) with STEMI. The overall mean age was 67.15 years (SD 12.88), and 29% of the population was male (*n* = 119). A total of 84 patients (20.5%) were of Jewish descent. The median Charlson Comorbidity Index (CCI) across the population was 3.0, with a range from 0 to 9, reflecting a wide range of chronic disease burdens. Ischemic heart disease was present in 92 patients (22.4%), diabetes mellitus in 40.4%, hypertension in 44.6%, and dyslipidemia in 60.5% of patients (Table 1a).

Most patients arrived via formal medical pathways: 52 patients (12.7%) ordered an ambulance themselves, 220 (53.7%) were referred by emergency centers, and 138 (33.7%) arrived independently. Late arrival was documented in 115 patients (28.0%). The overall median time from symptom onset to hospital arrival was 192.5 min [range, 12–4320 min], door-to-CT or needle was 54.5 min [5–450 min], and door-to-treatment (cardiac catheterization/thrombolysis or cerebral catheterization) was 67.0 min [9–365 min]. In-hospital mortality was 7.1%, and cumulative mortality rose gradually over time: 10.2% at 30 days, 13.9% at 90 days, 15.4% at 180 days, and 16.3% at one year. The median length of stay was 4.0 days [range, 0–40 days] (Table 1a,b).

Among stroke patients, all had documented treatment classification: 152 patients (70.0%) received no reperfusion therapy, 33 (15.2%) received systemic thrombolysis, 25 (11.5%) underwent cerebral catheterization, and seven patients (3.2%) received both interventions.

**Figure 1:**
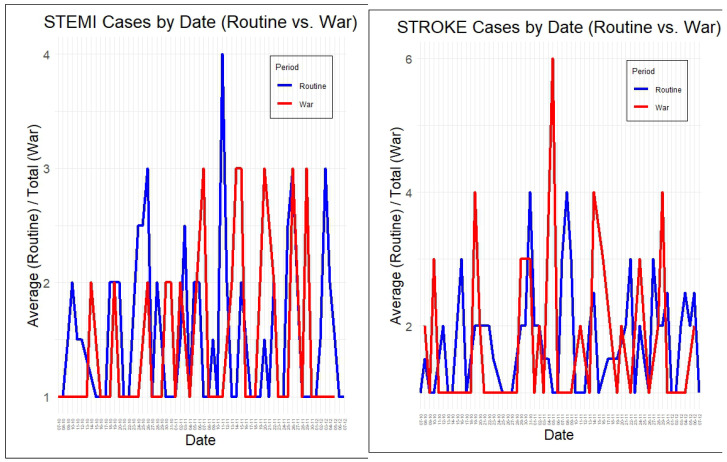
Daily stroke and STEMI cases during the study period. Line plots showing the daily counts of STEMI (**left**) and stroke (**right**) cases across the study period, separated by period type: routine (blue line) and war (red line). The x-axis represents the calendar date, and the y-axis shows either the total number of events (war) or the average count per day (routine), normalized to allow for visual comparison. Each point corresponds to the number of cases recorded on a specific day during the respective periods.

Figure 1 shows the distribution of daily cases during the study period. A notable reduction in STEMI admissions occurred during the first weeks of the war, which was followed by a rebound. In contrast, stroke admissions increased at the onset of the war and remained high throughout, suggesting different behavioral and clinical dynamics during the crisis. Significant results (*p*-value < 0.05) are bolded. 

### 3.2. Comparison Between Routine and War Periods (Within Diagnosis)

**Figure 2:**
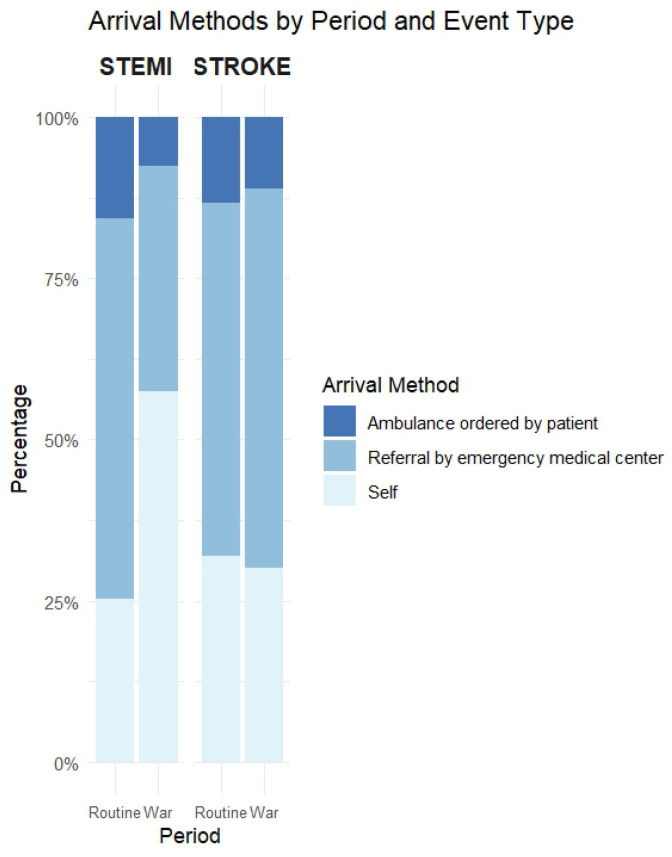
Stacked bar chart showing the distribution of arrival methods (self-arrival, referral by emergency medical center, or ambulance ordered by the patient) across four subgroups: STEMI during routine, STEMI during war, stroke during routine, and stroke during war. The x-axis denotes the event type and period, and the y-axis represents the percentage of patients in each arrival category. Each bar is segmented by arrival method and color-coded accordingly: light blue for self-arrival, medium blue for referral, and dark blue for ambulances.

**Figure 3:**
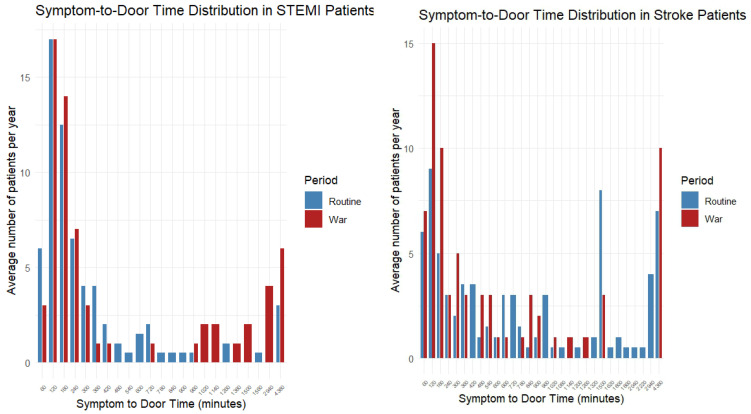
Time from symptoms to hospital arrival by Periods and Events. Bar plots showing the distribution of symptom-to-door (S2D) time in minutes among patients with STEMI (**left panel**) and stroke (**right panel**) during the routine (blue) and wartime (red) periods. The x-axis represents the S2D time intervals (in minutes), and the y-axis represents the average number of patients per year within each time interval. Each bar corresponds to a defined time range, and colors distinguish between the two periods.

**Figure 4:**
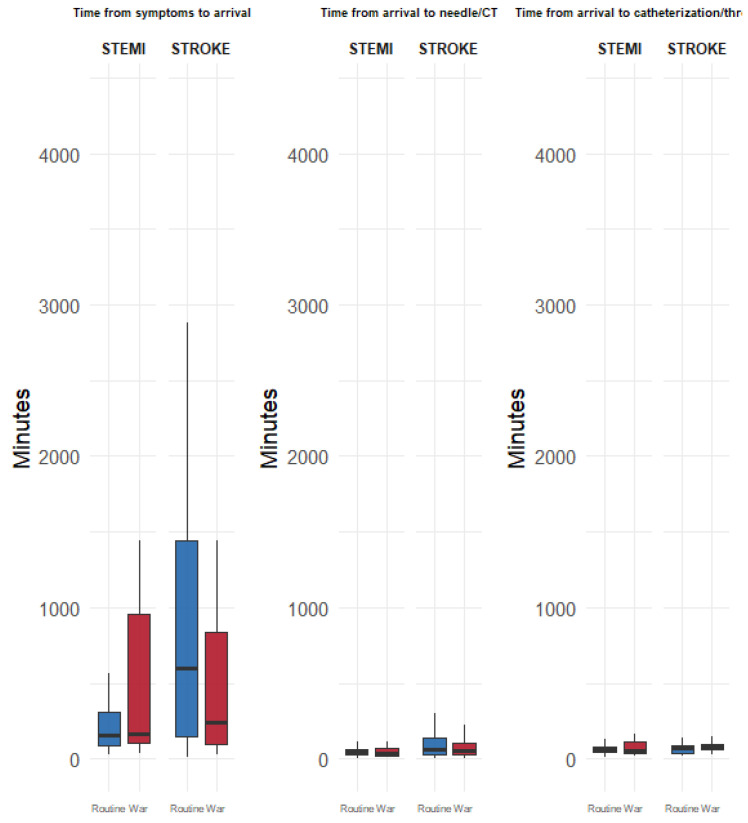
Timing of Periods and Events. Boxplots displaying the three time intervals for patients with STEMI and stroke during routine and wartime periods. From left to right: (1) time from symptom onset to hospital arrival, (2) time from arrival to needle/CT, and (3) time from arrival to catheterization/onset of definitive treatment. The x-axis categorizes event type (STEMI or stroke) and period (routine as blue or war as red), and the y-axis represents time in minutes. Each boxplot shows the median, IQR, and potential outliers.

**Figure 5:**
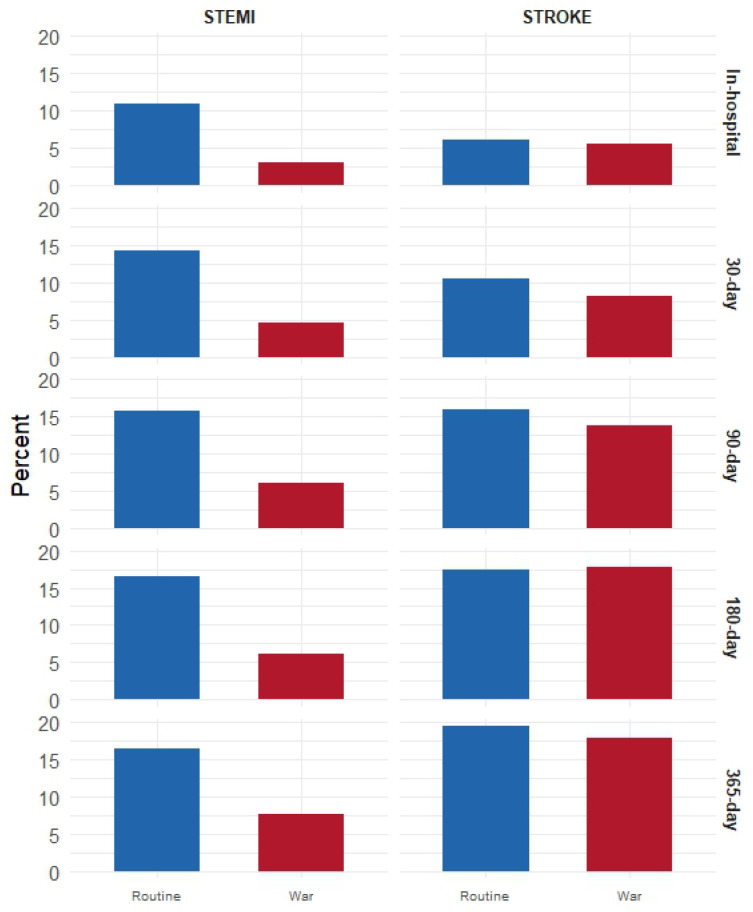
Mortality by Period and Event. Bar chart illustrating mortality percentages among STEMI and stroke patients across two periods (routine and war), stratified by follow-up duration: in-hospital, 30-day, 90-day, and 180-day mortality. The x-axis shows the period (routine in blue, war in red), and the y-axis represents percentage mortality. Separate panels are shown for STEMI (**left**) and stroke (**right**), with each mortality interval plotted as a horizontal bar group. Each bar corresponds to a specific time point and reflects the proportion of deceased patients within that subgroup.

In patients with STEMI, there were no statistically significant differences between the routine and war periods in terms of age (63.7 vs. 61.2 years, *p* = 0.169), male sex (19.7% vs. 10.6%, *p* = 0.16), Jewish ethnicity (23.6% vs. 24.2%, *p* = 1), or comorbidities (CCI, diabetes, hypertension, dyslipidemia; all *p* > 0.1) (Table 1a). However, during the war, patients arrived more often by self-transport (57.6% vs. 25.2%) and less often via referral or ambulance (*p* < 0.001) (Figure 2). Late arrivals were more frequent during the war period (28.8% vs. 10.2%, *p* = 0.002). There were no statistically significant differences in symptom-to-door time (Table 1b and Figure 3), door-to-needle and treatment time, mortality at any time point, or ICCU stay (Table 1b,c, Figure 4 and Figure 5).

Among patients with stroke, no significant differences were observed in age, sex, ethnicity, or comorbidities between the routine and war periods. Referral routes and ambulance use remained stable (Figure 2). Although late arrivals were less common during the war (30.1% vs. 42.4%, *p* = 0.109), the difference was not significant. Median symptom-to-door time decreased significantly from 600 to 246 min (*p* = 0.026) (Figure 3), whereas door-to-CT time slightly declined from 66 to 59 min, without reaching statistical significance (*p* = 0.186) (Figure 4). No differences were observed in in-hospital or cumulative mortality or ICCU stay duration (Figure 5).

### 3.3. Comparison Between Diagnoses (Stroke vs. STEMI)

During routine periods, patients with stroke were significantly older (71.0 vs. 63.7 years, *p* < 0.001), more likely to be male (37.5% vs. 19.7%, *p* = 0.002), and had a higher CCI (median 4.0 vs. 2.0, *p* < 0.001). Hypertension was more prevalent in stroke (70.1% vs. 43.3%, *p* < 0.001), whereas dyslipidemia was more common in STEMI (71.7% vs. 50.7%, *p* = 0.03). Patients with stroke experienced significantly longer median symptom-to-door times than those with STEMI (600 min [range, 12–4320] vs. 160 min [range, 29–4370], *p* < 0.001) (Figure 3) and longer median door-to-CT/needle times (66 vs. 50 min, *p* < 0.001) (Figure 4). No statistically significant differences were observed in mortality or ICCU stay (Table 1a,b).

During the war, similar differences in age (70.9 vs. 61.2 years, *p* < 0.001), male sex (45.2% vs. 10.6%, *p* < 0.001), and CCI (4.0 vs. 2.0, *p* < 0.001) were observed between patients with stroke and STEMI. Hypertension remained more common in stroke (75.3% vs. 56.1%, *p* = 0.026). Referral and arrival methods differed, with self-arrival being more common in STEMI (57.6% vs. 30.1%, *p* = 0.005) (Figure 2). No statistically significant differences were observed between diagnoses in terms of arrival timing, treatment delays, mortality, or LOS during the war (Table 1b,c; Figure 3, Figure 4 and Figure 5).

### 3.4. Adjusted Regression Models

**Figure 6:**
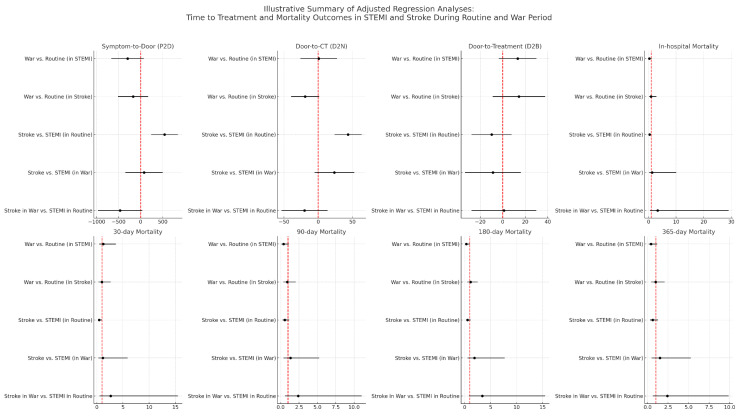
Illustrative summary of adjusted regression analyses: Time to treatment and mortality outcomes in STEMI and stroke during routine and war period. Forest plots depicting adjusted comparisons of time intervals and mortality endpoints between patients with STEMI and stroke, stratified by period (routine vs. war). Each subplot presents effect estimates (point estimate and 95% confidence intervals) for a specific outcome: time to treatment metrics (Symptom-to-Door, Door-to-CT, Door-to-Treatment) and mortality outcomes (in-hospital, 30-day, 90-day, 180-day, and 365-day). The x-axis in each plot represents the scale of the effect (minutes for treatment times, odds ratios for mortality), while the y-axis lists the specific pairwise contrasts. The vertical red dashed line denotes the null value (0 for linear models and 1 for odds ratio).

Multivariable regression models adjusted for age, sex, CCI, and arrival methods revealed that among patients with STEMI, wartime was associated with a trend toward lower 180-day mortality (OR = 0.33; 95% CI: 0.09–0.99; *p* = 0.07), although this difference did not reach statistical significance. Among patients with stroke, the war period was borderline significantly associated with reduced door-to-CT time (β = −19.05; 95% CI: (−39.42 to 1.31; *p* = 0.07), but showed no effect on treatment timing or mortality (Table 2 and Figure 6).

When comparing stroke to STEMI in the routine period, stroke was associated with significantly longer symptom-to-door (β = 543.07 min; *p* < 0.001) and door-to-CT times (β = 43.62 min; *p* < 0.001), and with lower odds of in-hospital (OR = 0.39; *p* = 0.05) and 30-day mortality (OR = 0.43; *p* = 0.04). These associations did not remain significant during the war, when the differences between stroke and STEMI disappeared. Comparing stroke during the war to STEMI during routine care showed no statistically significant differences in outcomes, although a trend toward higher 180-day mortality in stroke was observed (OR = 3.40; 95% CI: 0.87–15.48; *p* = 0.09) (Table 2 and Figure 6).

## 4. Discussion

### 4.1. Overview and Interpretation of Main Findings

This study provides a detailed side-by-side comparison of acute STEMI and ischemic stroke presentations during both wartime and routine periods, offering novel insights into diagnosis-specific responses to crisis conditions. While baseline demographics and comorbidity profiles remained stable, the wartime period was associated with notable shifts in arrival behavior, particularly among STEMI patients, who were more likely to arrive by self-transport and present later. In contrast, patients with stroke arrived more frequently and significantly earlier. Despite these behavioral disruptions, treatment timelines and outcomes remained largely unaffected, suggesting a degree of robustness in the system. However, the marked increase in self-transport among STEMI patients may reflect operational vulnerabilities or avoidance behaviors, and potential selection bias cannot be excluded. Patients with stroke consistently presented with older age, higher comorbidity burden, and longer delays, yet had lower short-term mortality compared to STEMI—a gap that narrowed during the conflict. These findings highlight both the adaptive capacity of emergency care pathways under duress and the distinct vulnerabilities of each condition, emphasizing the need for diagnosis-specific strategies for crisis preparedness.

### 4.2. Physiological, Logistical, and Behavioral Differences Between Diagnoses

The differences observed between STEMI and stroke are rooted in both pathophysiological and systemic factors. From a physiological perspective, STEMI symptoms—most commonly chest pain—are specific and immediately alarming, leading to quicker recognition and action [1,19]. In contrast, stroke symptoms are often ambiguous (e.g., dizziness, weakness, and confusion), potentially delaying recognition by patients or bystanders. Logistically, STEMI management in Israel typically involves direct EMS triage to catheterization labs based on prehospital ECG interpretation, often bypassing the ED and reducing in-hospital delays. However, stroke care generally requires neurologic assessment and imaging in the emergency department [20], introducing additional steps that contribute to consistently longer door-to-treatment times [21].

Behavioral and psychosocial factors likely contributed to these differences. Patients with STEMI —often younger and more mobile—were significantly more likely to arrive by self-transport during wartime, with a corresponding increase in late arrivals [22,23]. In contrast, patients with stroke presented more frequently and significantly earlier during wartime than during routine periods (median symptom-to-door time decreased from 600 to 246 min, *p* = 0.026). This pattern may reflect fear of exposure, transportation disruption, or mistrust of emergency services during conflict. In contrast, stroke patients, who are typically older and more dependent, may have relied on caregivers or community referrals, leading to more stable or improved access. The improvement in median symptom-to-door time for stroke patients during wartime (600 to 246 min, *p* = 0.026) supports this interpretation [7,24].

Psychosocial stress and demographic variations further influence these behaviors. Studies from other war zones, such as Lebanon and Ukraine, have reported that bombardment-related anxiety, socioeconomic instability, and psychological distress delay care-seeking and disproportionately affect vulnerable populations, including the elderly, ethnic minorities, and those with limited transportation or support networks [25,26]. Integrating psychosocial and demographic insights into emergency preparedness planning may enhance equity and effectiveness during future crises.

### 4.3. Biological Mechanisms and Conflict Comparison

The divergent trends observed during wartime—specifically, the increase in stroke admissions versus the decline in STEMI cases—may be partially explained by differential biological responses to acute stress. Activation of the hypothalamic–pituitary–adrenal axis and sympathetic nervous system induces transient hypertension, endothelial dysfunction, autonomic imbalance, and a pro-thrombotic state. These mechanisms disproportionately increase the risk of stroke, especially in individuals with hypertension or atrial fibrillation, both of which were more prevalent in our stroke cohort [27,28]. In contrast, STEMI is typically caused by atherosclerotic plaque rupture and thrombosis, a process that is less directly triggered by short-term neurohormonal fluctuations [29]. This biological plausibility is supported by studies of other conflicts, such as the Syrian civil war and the war in Bosnia and Herzegovina, where elevated cerebrovascular event rates were observed during periods of bombardment and displacement [30,31,32]. These parallels reinforce the role of stress-induced cerebrovascular vulnerability and support our findings.

### 4.4. Convergence of STEMI and Stroke Pathways During Wartime

A particularly striking observation is the convergence of care trajectories for STEMI and stroke during wartime. Although these conditions differ substantially in etiology and treatment pathways, both experience similar treatment times and outcomes under crisis conditions. This suggests that systemic pressures may have disproportionately disrupted STEMI care, which relies more heavily on early patient-initiated action and rapid EMS triage [5,22]. In contrast, stroke care, often triggered by third-party referrals and driven by centralized imaging protocols, was less affected [33]. Interestingly, the mortality advantage of stroke during routine times diminished during wartime conditions. This convergence may reflect both system-wide stress and the leveling of diagnosis-specific vulnerabilities.

The paradox of lower short-term mortality in stroke despite greater age and comorbidities may be explained by the differing mechanisms of early death. STEMI often results in immediate hemodynamic collapse due to pump failure or fatal arrhythmias, leading to a high concentration of deaths within the first 72 h [34,35]. In contrast, most ischemic strokes—particularly those not involving the brainstem or large hemispheric infarctions—are less likely to be acutely fatal. Reported in-hospital mortality rate for stroke ranges between 5% and 10%, with most early deaths resulting from secondary complications such as aspiration pneumonia, malignant cerebral edema, or hemorrhagic transformation [14,36,37,38]. These delayed mechanisms may account for the lower mortality observed in stroke than in STEMI. However, this gap narrowed during wartime, possibly due to increased physiological stress or systemic overload, which affected stroke outcomes more than in routine settings.

### 4.5. Comparison with Prior Literature

Our findings demonstrate both parallels and divergences from previously published studies on crisis-related disruptions in acute care. Similar to reports from the COVID-19 pandemic, we observed a decline in STEMI admissions and an increase in late presentation [5,22]. However, in contrast to studies showing stroke care deterioration during emergencies [7,24], our data revealed a relatively preserved—and even improved—stroke pathway during wartime. Most prior studies were conducted in the context of pandemics or natural disasters rather than active armed conflict.

While both situations induce acute public stress, the underlying mechanisms differ significantly. During the COVID-19 pandemic, care avoidance was largely driven by fear of infection and overwhelmed health systems, leading to voluntary delays despite an intact infrastructure [22,39]. In contrast, wartime conditions create direct physical access barriers, such as road closures, shelter-in-place orders, and security threats, which may limit mobility even when patients intend to seek care [40,41]. Furthermore, war-related trauma may provoke heightened vigilance for visible neurological deficits, potentially explaining the earlier arrival times in stroke patients. These behavioral and logistical distinctions may account for the contrasting trends observed in stroke care during crises.

The ability of SUMC to maintain treatment timelines for both diagnoses during continuous bombardment and regional instability distinguishes our setting from many others [14,42]. Factors such as centralized emergency protocols, dedicated personnel, and preexisting preparedness likely contributed to this apparent stability, although unmeasured factors such as differential care-seeking behavior may have influenced the observed outcomes [33,43]. Thus, our study contributes unique real-world evidence of the robustness of emergency care under military threat.

### 4.6. Practical and Policy Implications

Several actionable strategies have emerged from our findings. First, the high rate of self-transport and delayed arrival among patients with STEMI underscores the importance of preserving ambulance referral pathways during crises. Measures could include protected transport corridors or mobile ECG hubs [11]. Second, targeted public messaging campaigns for myocardial infarction symptoms may help reduce delays and improve patient outcomes [23,44]. Third, strengthening community preparedness through proactive public education, neighborhood-level engagement, and dissemination of clear emergency protocols can enhance resilience during crises. This includes campaigns to improve symptom recognition, guidance on when and how to seek emergency care, and leveraging trusted community figures to effectively deliver health messages. In high-risk areas, community-based drills and simulation exercises—conducted in collaboration with local emergency services—can improve public response and reduce hesitancy. Fourth, the relative stability of stroke care supports continued investment in centralized coordination, telestroke systems, and rapid imaging access [45]. Finally, emergency preparedness should include hospital-level drills simulating dual-diagnosis mass-casualty events to ensure balanced capacity across cardiovascular and neurologic emergencies [46].

### 4.7. Study Limitations

First, it was conducted at a single center in southern Israel and may not reflect patterns in other regions or healthcare systems [47]. Second, although data completeness was high, unmeasured confounders such as socioeconomic status, transportation access, and patient delay in symptom recognition were not captured [48]. Third, the sample size limited our ability to detect rare outcomes or perform subgroup analyses (e.g., by specific stroke subtype or revascularization method). Fourth, it is also possible that selection bias influenced our results, as high-risk patients—particularly those with severe functional impairment or limited access—may have avoided seeking care during wartime. This unmeasured confounding could have contributed to the observed stability in in-hospital outcomes and may have partially obscured hidden vulnerabilities in community-level care. Finally, although the wartime period analyzed included intense conflict, the findings may not be generalizable to other forms of crises, such as natural disasters or pandemics [49]. Nonetheless, the real-time warzone context and direct comparison of STEMI and stroke care provide robust, novel insights into healthcare crisis preparedness.

## 5. Conclusions

This study demonstrates that acute cardiovascular and neurologic emergencies exhibit diagnosis-specific vulnerabilities during armed conflict. While STEMI care was more susceptible to patient-initiated delays due to fear, transport barriers, and disrupted access, stroke care pathways remained relatively preserved, with a significant reduction in symptom-to-door time. These patterns suggest that stroke systems—anchored in centralized imaging and referral protocols—may offer greater structural consistency in times of crisis. In contrast, the reliance of STEMI care on rapid patient-initiated action and EMS triage underscores the need for tailored contingency plans. To improve system preparedness, centralized stroke protocols should be reinforced and implemented more broadly. Simultaneously, protected transport routes, mobile ECG units, and crisis-adapted public messaging can mitigate delays in STEMI care. These differentiated strategies can help ensure that emergency health services remain effective, equitable, and responsive—even under the extreme stressors of war.

## Figures and Tables

**Table 1:** (a): Demographic and clinical characteristics of patients with STEMI and stroke during the 2023 war and before the war. (b): Arrival characteristics and timelines of patients with STEMI and stroke during the 2023 war and before the war. (c) Outcomes of patients with STEMI and stroke during the 2023 war and before the war.

(**a**)												
		Entire population	Stroke				STEMI				Stroke vs. STEMI (*p*-value)	
		Overall	Overall	Routine	War	*p*-value	Overall	Routine	War	*p*-value	Routine	War
	n	410	217	144	73		193	127	66			
Demographic characteristics and background diseases	Male gender, n (%)		87 (40.1)	54 (37.5)	33 (45.2)	0.343	32 (16.6)	25 (19.7)	7 (10.6)	0.16	**0.002**	**<0.001**
	Age (mean (SD))	119 (29.0)	70.97 (12.53)	70.99 (12.78)	70.93 (12.12)	0.976	62.87 (11.90)	63.72 (11.97)	61.23 (11.69)	0.169	**<0.001**	**<0.001**
	Jew ethnicity, n (%)	67.15 (12.88)	38 (17.5)	23 (16.0)	15 (20.5)	0.516	46 (23.8)	30 (23.6)	16 (24.2)	1	0.152	0.75
	CCI (median [range])	84 (20.5)	4.00 [0.00, 7.00]	4.00 [0.00, 7.00]	4.00 [0.00, 6.00]	0.427	2.00 [0.00, 9.00]	2.00 [0.00, 9.00]	2.00 [0.00, 7.00]	0.23	**<0.001**	**<0.001**
	CIHD, n (%)	3.00 [0.00, 9.00]	49 (22.6)	34 (23.6)	15 (20.5)	0.735	43 (22.3)	24 (18.9)	19 (28.8)	0.166	0.426	0.352
	DM, n (%)	92 (22.4)	105 (48.4)	76 (52.8)	29 (39.7)	0.094	78 (40.4)	50 (39.4)	28 (42.4)	0.798	**0.037**	0.881
	HTN, n (%)	183 (44.6)	156 (71.9)	101 (70.1)	55 (75.3)	0.518	92 (47.7)	55 (43.3)	37 (56.1)	0.126	**<0.001**	**0.026**
	Dyslipidemia, n (%)	248 (60.5)	121 (55.8)	73 (50.7)	48 (65.8)	**0.049**	146 (75.6)	91 (71.7)	55 (83.3)	0.106	**<0.001**	**0.03**
	Previous stroke, n (%)		54 (24.9)	30 (20.8)	24 (32.9)	0.076						
(**b**)												
		Entire population	Stroke				STEMI				Stroke vs. STEMI (*p*-value)	
		Overall	Overall	Routine	War	*p*-value	Overall	Routine	War	*p*-value	Routine	War
	n	410	217	144	73		193	127	66			
Arrival characteristics	Arrival by, n (%)											
	Ambulance ordered by patient	52 (12.7)	27 (12.4)	19 (13.2)	8 (11.0)	0.825	25 (13.0)	20 (15.7)	5 (7.6)	**<0.001**	0.453	**0.005**
	Referral by an emergency medical center	220 (53.7)	122 (56.2)	79 (54.9)	43 (58.9)		98 (50.8)	75 (59.1)	23 (34.8)			
	Self	138 (33.7)	68 (31.3)	46 (31.9)	22 (30.1)		70 (36.3)	32 (25.2)	38 (57.6)			
	Late arrivals, n (%)	115 (28.0)	83 (38.2)	61 (42.4)	22 (30.1)	0.109	32 (16.6)	13 (10.2)	19 (28.8)	**0.002**	**<0.001**	1
Timelines	Time from symptoms to Arrival (median [range])	237.00 [12.00, 4370.00]	440.00 [12.00, 4320.00]	600.00 [12.00, 4320.00]	246.00 [30.00, 4320.00]	0.026	165.50 [29.00, 4370.00]	160.00 [29.00, 4370.00]	170.00 [40.00, 4320.00]	0.100	**<0.001**	0.656
	Time from arrival to needle/CT (median [range])	54.50 [5.00, 450.00]	63.00 [5.00, 450.00]	66.00 [5.00, 450.00]	59.00 [5.00, 355.00]	0.186	45.00 [7.00, 360.00]	50.00 [7.00, 270.00]	35.00 [10.00, 360.00]	0.385	**<0.001**	0.062
	Time from arrival to cardiac catheterization/thrombolysis or cerebral catheterization (median [range])	67.00 [9.00, 365.00]	72.00 [20.00, 184.00]	69.00 [20.00, 163.00]	84.00 [30.00, 184.00]	0.116	63.00 [9.00, 365.00]	67.50 [9.00, 280.00]	52.00 [20.00, 365.00]	0.457	0.925	0.099
(**c**)												
		Entire population	Stroke				STEMI				Stroke vs. STEMI (*p*-value)	
		Overall	Overall	Routine	War	*p*-value	Overall	Routine	War	*p*-value	Routine	War
	n	410	217	144	73		193	127	66			
Outcomes	In hospital death, n (%)	29 (7.1)	13 (6.0)	9 (6.2)	4 (5.5)	1	16 (8.3)	14 (11.0)	2 (3.0)	0.102	0.449	0.771
	Death within 30 days, n (%)	42 (10.2)	21 (9.7)	15 (10.4)	6 (8.2)	0.784	21 (10.9)	18 (14.2)	3 (4.5)	0.073	1	0.593
	Death within 90 days, n (%)	57 (13.9)	33 (15.2)	23 (16.0)	10 (13.7)	0.81	24 (12.4)	20 (15.7)	4 (6.1)	0.088	0.985	0.226
	Death within 180 days, n (%)	63 (15.4)	38 (17.5)	25 (17.4)	13 (17.8)	1	25 (13.0)	21 (16.5)	4 (6.1)	0.067	0.644	0.064
	Death within 365 days, n (%)	67 (16.3)	41 (18.9)	28 (19.4)	13 (17.8)	0.914	26 (13.5)	21 (16.5)	5 (7.6)	0.132	0.449	0.123
	LOS (median [range])	4.00 [0.00, 40.00]	5.00 [0.00, 40.00]	6.00 [0.00, 40.00]	5.00 [0.00, 24.00]	0.241	4.00 [1.00, 15.00]	4.00 [1.00, 15.00]	4.00 [1.00, 9.00]	0.834	**<0.001**	**0.005**

**Table 2:** Effect of war vs. routine period and event type (STEMI vs. Stroke) on clinical outcomes: adjusted regression estimates.

	P2D (Symptom to Door)	D2N (Door to CT)	D2B (Door to Treatment)	In-Hospital Death	30-Day Mortality	90-Day Mortality	180-Day Mortality	365-Day Mortality
War vs. Routine (in STEMI)	**−295.42 (−665.34, 74.50); *p* = 0.117**	0.88 (−25.81, 27.57); *p* = 0.94	13.24 (−3.55, 30.03); *p* = 0.12	0.27 (0.04, 1.04); *p* = 0.10	1.23 (0.47, 3.65; *p* = 0.68	0.38 (0.10, 1.12); *p* = 0.10	0.33 (0.09, 0.99); *p* = 0.07	0.41 (0.12, 1.16); *p* = 0.11
War vs. Routine (in Stroke)	−171.38 (−513.17, 170.42); *p* = 0.325	**−19.05 (−39.42, 1.31); *p* = 0.07**	14.33 (−9.01, 37.67); *p* = 0.23	0.88 (0.23, 2.86); *p* = 0.85	0.96 (0.32, 2.63); *p* = 0.94	0.89 (0.37, 2.04); *p* = 0.79	1.15 (0.51, 2.50); *p* = 0.74	0.98 (0.44, 2.08); *p* = 0.97
Stroke vs. STEMI (in Routine)	**543.07 (239.68, 846.47); *p* < 0.001**	**43.62 (23.94, 63.30); *p* < 0.001**	−9.79 (−27.55, 7.98); *p* = 0.28	**0.39 (0.15, 0.97); *p* = 0.05**	**0.43 (0.19, 0.94); *p* = 0.04**	0.55 (0.26, 1.14); *p* = 0.11	0.56 (0.27, 1.14); *p* = 0.11	0.62 (0.30, 1.26); *p* = 0.18
Stroke vs. STEMI (in War)	76.28 (−349.84 502.40); *p* = 0.725	23.69 (−5.14, 52.52); *p* = 0.11	−8.69 (−33.46, 16.08); *p* = 0.49	1.27 (0.22, 10.00); *p* = 0.80	1.14 (0.27, 5.88); *p* = 0.87	1.32 (0.38, 5.26); *p* = 0.68	1.89 (0.57, 7.69); *p* = 0.32	1.49 (0.47, 5.26); *p* = 0.52
Stroke in War vs. STEMI in Routine	−466.79 (−971.25, 37.67); *p* = 0.07	−19.93 (−53.56, 13.71); *p* = 0.24	1.09 (−27.65, 29.83); *p* = 0.94	3.27 (0.48, 29.07); *p* = 0.24	2.64 (0.53, 15.49); *p* = 0.25	2.37 (0.58, 11.02); *p* = 0.24	3.40 (0.87, 15.48); *p* = 0.09	2.40 (0.64, 9.90); *p* = 0.21

Significant results (*p*-value < 0.05) are bolded.

## Data Availability

According to the ethics committee, the data cannot be made available due to concerns regarding patient confidentiality. However, data may be made available upon reasonable request and are subject to specific approval by the ethics committee. Requests for data access can be directed to Zeldetz and will be reviewed in accordance with ethical guidelines and institutional policies.

## Abbreviations

Abbreviation	Full Term
STEMI	ST-Elevation Myocardial Infarction
ED	Emergency Department
EMS	Emergency Medical Services
CT	Computed Tomography
ICCU	Intensive Cardiac Care Unit
CCI	Charlson Comorbidity Index
P2D	Symptom-to-Door Time
D2N	Door-to-Needle (for STEMI)/Door-to-CT (for stroke)
D2B	Door-to-Balloon (for STEMI)/Door-to-Treatment
SUMC	Soroka University Medical Center
SD	Standard deviation
OR	Odds Ratio
CI	Confidence Interval
LOS	Length of Stay
